# Editorial: Self-directed Prevention and Recovery: E-health Interventions in Addiction Science

**DOI:** 10.3389/fpsyt.2021.844859

**Published:** 2022-02-01

**Authors:** Máté Kapitány-Fövény, Stephanie Carreiro, Grant Christie

**Affiliations:** ^1^Faculty of Health Sciences, Semmelweis University, Budapest, Hungary; ^2^National Institute of Mental Health, Neurology and Neurosurgery Nyíro Gyula Hospital, Budapest, Hungary; ^3^Division of Medical Toxicology, Department of Emergency Medicine, University of Massachusetts Chan Medical School, Worcester, MA, United States; ^4^Department of Psychological Medicine, Faculty of Medical and Health Sciences, The University of Auckland, Auckland, New Zealand

**Keywords:** e-health, addiction science, digital health, treatment modalities, efficacy

Addictive disorders represent a significant global burden and suffer from high rates of untreated patients (e.g., ~90% in case of alcohol use disorder) due to issues such as stigma, time restraints, financial barriers, or the limited number of addiction services. E-health and mHealth, related concepts which represent the intersection between electronic technologies, communication and all facets of healthcare, are particularly attractive options to reach and help this hidden population. In the past 2 years, mitigation strategies to decrease the spread of COVID-19, including complete or partial lockdowns, have further highlighted the benefits of telemedicine and other digital health tools, and have sped up the introduction of novel ways of supporting clients. A variety of engagement, assessment, and treatment processes can be conducted virtually or via e-health including screening and brief interventions, psychoeducation, symptom rating, substance use monitoring, goal setting, and other CBT strategies and relapse prevention to name a few. Novel ways to monitor and predict risk are emerging from new technologies such as wearable sensors and artificial intelligence.

The advantages of e-health programs are wide ranging. These include (1) providing anonymity for their target groups, (2) ensuring therapeutic access in regions where addiction care is not available, (3) eliminating the hierarchical relationship between the care provider and the client, (4) increasing the effectiveness of self-management, and (5) empowering clients to be in control of their own recovery journey. In view of the rapidly expanding body of research in this field, it is timely and important to provide an overview on the state-of-the-art, efficacy (including the benefits and limitations), and feasibility of such programs. In addition, perspectives on the attitudes toward e-health interventions from both clients and treatment providers across the range of substance use disorders (SUD), both in the general population and among special subpopulations as well (e.g., pregnant women with SUD), are essential.

Prior literature supports the promise of e-health for mental health disorders, and specifically SUDs. However, several gaps in knowledge have been identified, including efficacy, implementation, and the integration of sensors for continuous data acquisition. We invited submissions from scholars in the field of addiction research to fill these gaps. The current Research Topic contains seven papers that cover the following major topics:

- Review of the currently available e-interventions for SUD.
- The usability, acceptability and effectiveness of e-Health interventions in relapse prevention and the management of SUD.
- Novel analytical methods in the prediction of SUD problem severity and clinical course.
- Evaluations of subpopulations, including pregnant women and specific substance use disorders (i.e. cannabis and alcohol use disorder).

The primary goal of the current article collection is to disseminate knowledge with researchers and clinical practitioners across a broad range of e-health concepts in addiction science, examining applied methodologies, current data and barriers in terms of efficacy, availability, and cost-effectiveness. As the theoretical and empirical basis for the development, implementation and evaluation of more efficacious e-health programs in the addiction field progresses, considerations related to disease or substance specific approaches (e.g., interventions targeted toward AUD vs. SUD in general), treatment modalities (mobile app, web, informatics, or sensor based approaches) and the optimal role in clinical care (digital diagnostic vs. digital therapeutic) will become increasingly important.

## Author Contributions

MK-F, SC, and GC collaborated in writing the Research Topic proposal, sending submission invitations to scholars in the field of addiction research, checking the eligibility of submitted abstracts, and assigning each submissions to reviewers. The overall process was monitored by all three topic editors. All authors contributed to the article and approved the submitted version.

## Funding

MK-F acknowledges the support by the János Bolyai Research Scholarship of the Hungarian Academy of Sciences and the support by the ÚNKP-21-5 New National Excellence Program of the Hungarian Ministry for Innovation and Technology. SC acknowledges the funding support from NIH/NIDA Grant (No. K23DA045242).

## Conflict of Interest

The authors declare that the research was conducted in the absence of any commercial or financial relationships that could be construed as a potential conflict of interest.

## Publisher's Note

All claims expressed in this article are solely those of the authors and do not necessarily represent those of their affiliated organizations, or those of the publisher, the editors and the reviewers. Any product that may be evaluated in this article, or claim that may be made by its manufacturer, is not guaranteed or endorsed by the publisher.

